# Foliar Cellulose and Lignin Degradation of Two Dominant Tree Species in a Riparian Zone of the Three Gorges Dam Reservoir, China

**DOI:** 10.3389/fpls.2020.569871

**Published:** 2020-12-07

**Authors:** Zhangting Chen, Xuemei Chen, Chaoying Wang, Changxiao Li

**Affiliations:** ^1^Key Laboratory of Eco-Environments in the Three Gorges Reservoir Region (Ministry of Education), State Cultivation Base of Eco-agriculture for Southwest Mountainous Land, College of Life Sciences, Southwest University, Chongqing, China; ^2^School of Tourism Management, Guilin Tourism University, Guilin, China; ^3^Chongqing City Management College, Chongqing, China

**Keywords:** cellulose, lignin, degradation, Three Gorges Dam Reservoir, water treatment

## Abstract

The riparian zone can affect the degradation of foliar cellulose and lignin by changing the hydrological gradient of the foliage decomposition environment. However, their degradation dynamics during the process of foliage decomposition remain unclear in mixed plantation forests in a riparian zone. Herein, we explored degradation of cellulose and lignin for two dominant riparian species, bald cypress [*Taxodium distichum* (L.) Rich.] and Chinese willow (*Salix matsudana* Koidz.), and a combined treatment with equal proportions of foliar mass of these species, involving three water treatments [no submergence (CK), shallow submergence (SS), and deep submergence (DS)] in a riparian zone of the Three Gorges Dam Reservoir (TGDR), China. Throughout an entire year’s incubation, the degradation of cellulose and lignin was 55.57–97.76% and 79.87–93.82%, respectively. In the early stage of decomposition (i.e., the first 30 days), cellulose and lignin were rapidly degraded, and the mass loss of cellulose and lignin in water environments (SS and DS) was greater than that in a non-flooded environment (CK) regardless of the foliage type. The degradation of cellulose and lignin was mainly related to the quality of the leaf litter (as indicated by the concentrations of cellulose and lignin, and the contents of C, N, and P), decomposition period, and local environmental factors (temperature, water gradients, and dissolved oxygen). Our results will provide a clear insight into the material cycling process in a riparian zone of the TGDR and similar ecosystems in other regions.

## Introduction

Riparian zones serve as crucial domains in watershed ecosystems, since they form transitional zones for the transfer of nutrients and the flow of energy between terrestrial and aquatic ecosystems (González et al., 2017; Zhang et al., 2020). The decomposition of allochthonous organic materials (e.g., leaf litter) in riparian zones is vital in providing for the persistent availability of both nutrient and energy resources in these dynamic riparian environments (Edmonds and Tuttle, 2010; Britson et al., 2016). When compared with those in terrestrial environments, the effects of scouring and leaching are stronger in aquatic environments, which increase the physical fragmentation of leaves, which can then decompose much more rapidly in riparian environments than on forest floors (Lecerf et al., 2007; Graça et al., 2015). Because plant litter is mainly composed of carbohydrate polymers (cellulose and hemicellulose) and aromatic macromolecules (lignin), the availability of nutrients and energy in the riparian zones is subject to the degradation of cellulose and lignin in riparian litter (Berg and McClaugherty, 2014). However, few data are available on the dynamics of the relatively rapid decomposition of cellulose and lignin in leaf litter that occurs in riparian zones. Thus, this prevents us from having a more-in-depth understanding of the changes in foliar cellulose and lignin degradation, as well as of the nutrient transportation and energy flow in riparian ecosystems.

Cellulose is among the most readily available source of carbon (C) in plant litter (Berg and McClaugherty, 2014), followed by lignin, and it accounts for about 30% of the C in plants (Boerjan et al., 2003); the degradation of cellulose contributes significantly to C fluxes during litter decomposition and also exerts significant control on the litter decomposition process (Schwarz, 2001). In the foliage decomposition process, cellulose degrades more quickly than lignin does, so the degradation of cellulose is believed to play a dominant role during the earlier stages of foliage decomposition (Berg and McClaugherty, 2014; He et al., 2015). In contrast, lignin is the main component of the cell walls of vascular plants, and its chemical composition and structural characteristics give it high chemical stability and resistance to microbial degradation (Kogel-Knabner, 2002; Janusz et al., 2017). Therefore, lignin is a recalcitrant constituent of the litter substrate (Talbot et al., 2012; Janusz et al., 2017). Furthermore, lignin forms a physical barrier that slows enzymatic hydrolysis of most cellulose; therefore, cellulose cannot decompose independently of lignin (Cooke and Whipps, 1993), and cellulose may degrade much more rapidly in water than on a forest floor as a result of the earlier degradation of lignin in a river. However, past research studies on the degradation process of refractory substances such as cellulose and lignin in leaf litter have mostly been conducted in forest habitats (He et al., 2015; Li et al., 2016; He et al., 2019), while few studies have been conducted in riparian zones. Nevertheless, studying the degradation of cellulose and lignin in leaf litter within the context of riparian environments is of great significance as it helps researchers to understand the nutrient cycling process of riparian ecosystems and hence supports the sustainable management of such land-water ecotones.

One of the main factors affecting foliage decomposition is the type of species involved (Makkonen et al., 2012); the degradation rates of cellulose and lignin can vary greatly among different foliage types (Fioretto et al., 2005). Moreover, the heterogeneity of species composition can additionally interact with the microclimate to change the local decomposition environment, affecting the degradation of foliar cellulose and lignin (Thevenot et al., 2010; Li et al., 2016). Local environmental factors may directly (e.g., temperature, moisture, and dissolved oxygen) and indirectly (e.g., through affecting the decomposer community) affect the process of foliage decomposition (Berg and McClaugherty, 2014). Furthermore, decomposition of the foliage of these species in the riparian zone is not only regulated by similar factors in terrestrial ecosystems (such as climate, substrate quality, and availability of environmental nutrients) (Cornwell et al., 2008; Gessner et al., 2010), but it is also affected by the unique environmental factors of the riparian zone, such as the relatively stable temperature, sufficient water sources, low oxygen, and strong scouring effects (Zhang and Lou, 2011; Xu et al., 2013; Yuan et al., 2013). Moreover, the depth of submergence and its dynamics in the riparian zone can influence decomposition events in the field (Wallis and Raulings, 2011; Xie et al., 2015; Martínez et al., 2016); these may also indirectly affect the process of decomposition. Thus, the physical and chemical properties of leaf litter and local environmental factors should be simultaneously considered when studying the degradation of foliar cellulose and lignin of the riparian zone. However, the effects of environmental factors such as hydrological gradients on foliar cellulose and lignin degradation in the riparian zone have been rarely reported to date.

After completion of the Three Gorges Dam Reservoir (TGDR) in China, a water-level fluctuation zone covering an area of 349 km^2^ was created with an annual periodic water level change (Yuan et al., 2013). Such a dynamic hydrological regime in the TGDR has created a great challenge to understanding the process of nutrient cycling and energy flow associated with decomposition of plants, especially as it is related to the degradation of foliar cellulose and lignin (because of its total volume), in this newly created vast riparian zone. For channel safety, all the trees in water-level fluctuation zone of the TGDR were planted at or 165 m above sea level; although these plants have a strong ability to endure long-term flooding, nevertheless, they are inevitably subjected to long-term deep submergence annually, thereby generating large amounts of litter. In order to better understand the degradation dynamics of cellulose and lignin in riparian ecosystems, we conducted a one-year (i.e., one hydrological cycle) experiment *in situ*, involving two dominant riparian tree species [bald cypress: *Taxodium distichum* (L.) Rich.; Chinese willow: *Salix matsudana* Koidz.] and their mixtures (samples involving mixtures of the foliage from these two species in equal mass proportions) under three water treatments [no submergence (CK), shallow submergence (SS), and deep submergence (DS)]. We focused on determining (1) how water treatments affect the degradation of cellulose and lignin of different foliage types and (2) whether degradation rates vary between different foliage types. Considering that foliage can decompose faster in riparian zone, we hypothesized that (1) both cellulose and lignin may degrade in the earlier of foliage decomposition process and (2) the degradation rates of cellulose and lignin may vary with different foliage types and local environment in which decomposition takes place. The results of the study will offer theoretical recommendations for the degradation of recalcitrant substances in reforestation tree species in the TGDR riparian zone and other similar regions, as well as the scientific plantation management of planted forests.

## Materials and Methods

### Study Site

The *in situ* experiment was conducted in a revegetation demonstration site next to the Ruxi River of Zhong County, Chongqing Municipality, China (30°24′16–56″N; 108°08′03–21″E). The Ruxi River is located in the heart of the TGDR area and is one of the important first-level tributaries in the TGDR (Tang et al., 2015). The local area has abundant rainfall, adequate sunshine, a long frost-free period, and a southeastern subtropical climate (Qin and Gao, 2004). The reforestation trees are dominated by bald cypress and Chinese willow, including both monocultures and mixed plantations. The changing water level of Ruxi River, a tributary of the TGDR, fluctuates dynamically throughout the year; therefore, we conducted the foliage decomposition experiment in a man-made reservoir next to the Ruxi River to effectively control the water treatments during the experiment period. This reservoir is 250 m long, 60 m wide, and 12 m deep and covers an area of more than 700 m^2^, with its water being impounded directly from the Ruxi River.

### Experimental Design

Bald cypress and Chinese willow are two dominant species used in riparian restoration of the TGDR area and have been widely planted because of their strong tolerance to inundation. Forest managers often use a mixed planting of coniferous and broad-leaved trees to increase species diversity, prevent soil erosion, and ensure the sustainable development of plantations (Li et al., 2010; Wang et al., 2016); a mixed species plantation provides a good measure that can be used to protect the environment of riparian ecosystems (Schoonover et al., 2006).

In the riparian zone of the TGDR, the plant leaves from a variety of perennial woody species are submerged when the water level rises in October each year. The special hydrological gradient of the TGDR results in the leaves of these flood-resistant trees still being present on the branches when the water level rises, and so at the time that they are flooded, the leaves are fresh; however, they will be shed and inevitably rot under long-term submergence, completing the whole decomposition process as litter. Therefore, our study was designed based on the actual situation of the leaves being flooded; fresh leaves were selected as experimental materials. We used the litterbag method to conduct the experiment, which was synchronized with the actual fluctuation of the water level in the TGDR. On September 2017, fresh leaves of bald cypress and Chinese willow were collected from 12 trees with the same growth conditions; we placed 15-g samples of foliage type in each litterbag (20 × 20 cm with a 0.25-mm mesh size). We put three foliage types in the litterbags: 15 g of pure bald cypress, 15 g of pure Chinese willow, and combinations consisting of two 7.5-g subsamples of each species (bald cypress + Chinese willow). The litterbags of each foliage type were then randomly divided into three groups for the different water treatments as follows: CK, as a control check, meaning that the sample decomposed on the ground throughout the year without being flooded; SS, 0.5-m shallow water submergence, where the sample decomposed at a depth of 0.5 m in water throughout the year; DS, 5-m-deep water submergence, where the sample decomposed at a depth of 5 m in water throughout the year. We placed the litterbags in the field on 25 September 2017 and removed it on 26 September 2018 after a one-year (i.e., one hydrological cycle) submergence or exposure. Ten sampling dates were selected; we prepared 120 replications for each of the type combinations to monitor the dynamics of foliage decomposition and cellulose and lignin degradation.

### Sample Analysis

On the day of each sample recovery, the samples were gently rinsed to remove sediments or invertebrates, dried in the oven at 65°C for 72 h, and then weighed in the laboratory. After the subsamples were ground and sieved them through a 0.3-mm sieve, the concentrations of cellulose and lignin were analyzed by the widely used acid detergent lignin method, as previously described (Graça et al., 2005). An additional 12 samples of the three foliage types were prepared to assess the initial dry weight and nutrient content (Table 1). Across the experiment period, the air and water temperature, and the dissolved oxygen content of the experimental reservoir were concurrently recorded at each sampling date using a Hydrolab DS5 water quality multiparameter monitor (Hydrolab-Hach Corp., Loveland, CO, United States) (Table 2).

**TABLE 1:** Initial foliage chemical characteristics of bald cypress, Chinese willow, and the mixed environments.

Foliage type	C (%)	N (%)	P (%)	Cellulose (%)	Lignin (%)	C/N	C/P	N/P	Lignin/N
Bald cypress	47.04	1.61	0.26	15.77	36.43	28.86	187.32	6.26	2.27
	(2.82)^a^	(0.13)^c^	(0.03)^a^	(2.4)^b^	(1.98)^a^	(0.53)^a^	(4.39)^b^	(0.11)^c^	(0.14)^a^
Chinese willow	42.39	2.01	0.20	21.65	23.23	20.09	209.75	10.23	1.11
	(0.34)^c^	(0.20)^a^	(0.03)^b^	(1.08)^a^	(2.51)^b^	(0.18)^c^	(2.07)^a^	(0.21)^a^	(0.12)^b^
Mixtures	45.01	2.00	0.21	20.53	24.88	22.92	213.97	9.31	1.26
	(0.84)^b^	(0.36)^b^	(0.03)^b^	(0.83)^ab^	(2.65)^b^	(0.58)^b^	(3.57)^a^	(0.16)^b^	(0.83)^b^

*Values represent means with standard deviations in parentheses (*n* = 12). Different lowercase letters indicate a significant difference between foliar type for the same variable at *P* < 0.05. C, total organic carbon; N, total nitrogen; P, total phosphorus.*

**TABLE 2:** Mean values for the characteristics of the local environmental variables in different water treatments during the one-year experimental period.

Index	Water treatment		
	CK	SS	DS
AT (°C)	23.61 (1.12)	21.64 (1.25)	15.66 (0.62)
pH	6.16 (0.04)	6.7 (0.05)	6.89 (0.07)
WC (μS⋅m^–1^)	409.25 (5.55)	406.16 (7.83)	402.23 (9.05)
DO (mg⋅L^–1^)	0	15.24 (0.46)	5.7 (0.41)

*CK, SS, and DS involve no submergence, shallow submergence, and deep submergence, respectively. Values represent the means with standard deviations in parentheses across the one-year experiment (*n* = 120). AT, average temperature; WC, water conductivity; DO, dissolved oxygen.*

### Statistical Analysis

The degradation rate *k* of cellulose and lignin was calculated by exponential decay model (Olson, 1963).

The mass remaining (*W*) and degradation rate (*L*) of cellulose and lignin in each phase during decomposition were calculated as follows:

$${\text{W}}_{\text{t}}=M_{\text{t}}\times {\text{C}}_{\text{t}}$$

${\text{L}}_{\text{t}}\left(\%\right)=\frac{{\text{M}}_{\text{t}-1}\,{\text{C}}_{\text{t}-1}-M_{\text{t}}\,{\text{C}}_{\text{t}}}{{\text{M}}_{0}\,{\text{C}}_{0}\,\Delta \,{\text{T}}_{\text{t}}}\times 100\%$ (t = 1, 2, 3, 4, 5, 6, 7, 8, 9, 10), where *M*_t_ and M_t__–__1_ are the remaining mass litter at sampling times t and t − 1, respectively; *C*_t_ and *C*_t__–__1_ are the concentrations of cellulose or lignin at sampling time *t* and t − 1, respectively; *M*_0_ and *C*_0_ are the initial dry mass and cellulose and lignin concentrations; and Δ*T*_t_ is the decomposition time in months between the sampling time t − 1 and t (Yue et al., 2016).

We used a repeated-measures analysis of variance (ANOVA) to examine the effects of leaf type, decomposition period, water treatment, and the interaction of two or three of them on degradation of cellulose and lignin across the one-year experiment. We performed a one-way ANOVA to evaluate the effects of decomposition period and each foliage type independently in exploring the variations in mass, concentration, and cellulose and lignin degradation rate during different decomposition time for each foliage type. Where the ANOVA results was significant (*P* < 0.05), we determined the differences among means using a Duncan honestly significant difference test. In addition, the stepwise linear regression analysis was used to examine the principal element (changes in chemical traits of samples) that affected the cellulose and lignin degradation rates in different water treatments. All analyses were performed in SPSS 22.0 (IBM Corp., Chicago, IL, United States) and Origin 8.5 (Origin Lab Crop., Northampton, MA, United States). Herein, we show data as mean ± standard error (SE).

## Results

### Foliage Mass Loss, and Cellulose and Lignin Mass Remaining

**FIGURE 1:**
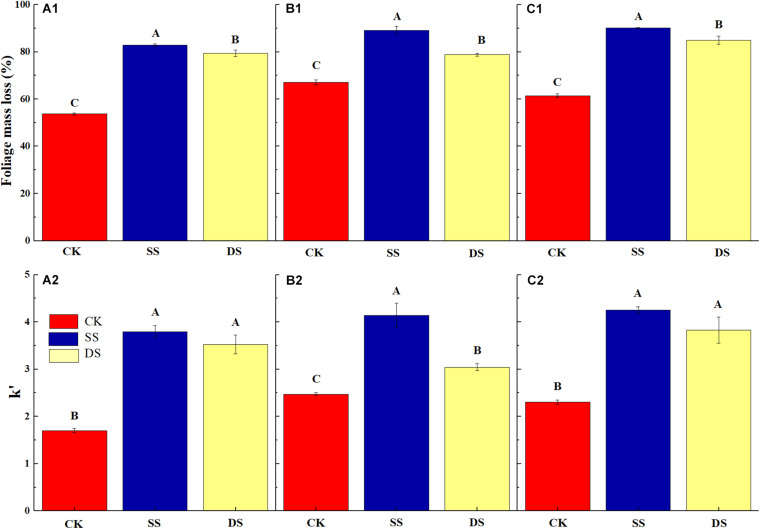
The mass loss (%) of bald cypress **(A1)**, Chinese willow **(B1)**, and the mixtures **(C1)** at the end of the one-year experiment; and the decomposition rate (k′, k × 10^3^) of bald cypress **(A2)**, Chinese willow **(B2)**, and the mixtures **(C2)** during the decomposition period. Different uppercase letters indicate significant (*P* < 0.05) differences of mass loss as well as decomposition rate for each foliage type; CK, SS, and DS represent no submergence, shallow submergence, and deep submergence, respectively.

**FIGURE 2:**
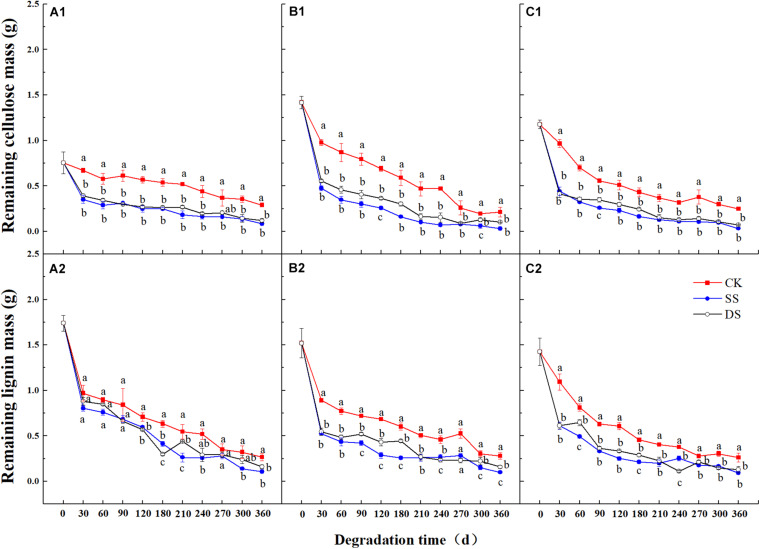
The remaining mass of cellulose and lignin of bald cypress **(A1,A2)**, Chinese willow **(B1,B2)**, and the mixtures **(C1,C2)** during each decomposition period across one-year. Different lowercase letters indicate significant (*P* < 0.05) differences of cellulose and lignin mass for each foliage type among different submergence depths at each sampling time; CK, SS, and DS represent no submergence, shallow submergence, and deep submergence, respectively.

Over the one-year decomposition period, the three foliage types lost 83–90% of their initial dry mass in SS and 79–85% in DS, compared with 54–67% in CK. Regardless of the foliage type, the foliage mass loss was the greatest with the SS water treatment, followed by DS, and the CK had the least (Figure 1). With the foliage decomposition, the remaining mass of cellulose and lignin from each foliage type decreased continuously (Figure 2), but there were significant differences between foliage type (except for lignin degradation), water treatment, and decomposition period (*P* < 0.05) (Table 3). Similar to the mass loss pattern, at the end of the decomposition period, the remaining mass of cellulose and lignin of the CK treatment was significantly greater than that of SS and DS treatments (*P* < 0.05).

**TABLE 3:** Results of repeated measurement analysis of foliage type, decomposition time, submergence depth, and their interaction on cellulose and lignin degradation rates (%/month) over the one-year experiment.

Factor	*d.f.*	Cellulose		Lignin	
		*F-*value	*P*-value	*F*-value	*P*-value
Foliage type	2	24.07	***	0.310	ns
Submergence depth	2	41.86	***	20.116	***
Time	9	99.05	***	207.559	***
Foliage type × Submergence depth	4	1.04	ns	0.663	ns
Foliage type × Time	18	1.58	ns	6.188	***
Submergence depth × Time	18	3.30	**	3.415	**
Foliage type × Submergence depth × Time	36	0.86	ns	2.460	**

d.f., degrees of freedom; ns, P > 0.05; *P < 0.05; **P < 0.01; ***P < 0.001.

### Concentration of Cellulose and Lignin

**FIGURE 3:**
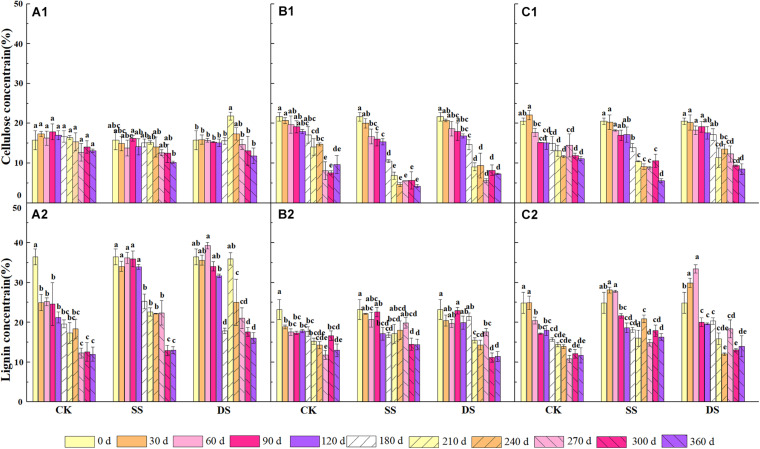
The concentrations of cellulose and lignin of bald cypress **(A1,A2)**, Chinese willow **(B1,B2)**, and the mixtures **(C1,C2)** during each decomposition period across one-year. Different lowercase letters indicate significant (*P* < 0.05) differences of cellulose and lignin concentration among different submergence depths at each sampling time for each foliage type. CK, SS, and DS represent no submergence, shallow submergence, and deep submergence, respectively.

The cellulose concentration continued to decrease throughout the decomposition process but differed among the foliage types and the water treatments (*P* < 0.05) (Figure 3). At the end of the experiment, all foliage types showed the lowest concentration of cellulose in the SS treatment, followed by DS and CK treatments. The cellulose concentration of Chinese willow was the lowest among the three water treatments by the end of decomposition. The lignin concentration was different from the cellulose, which differed greatly under different foliage types and water treatments. However, both of the concentrations of the cellulose and lignin remained significantly lower than the initial values after one-year of decomposition (*P* < 0.05).

### Degradation Rates of Cellulose and Lignin

**FIGURE 4:**
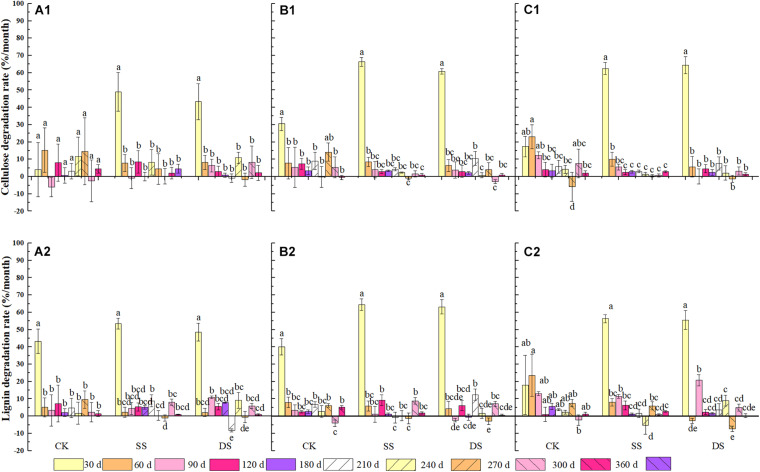
The degradation rate of cellulose and lignin of bald cypress **(A1,A2)**, Chinese willow **(B1,B2)**, and the mixtures **(C1,C2)** during each sampling time across one-year. Different lowercase letters indicate statistically significant (*P* < 0.05) differences of cellulose and lignin degradation rate among different submergence depths at each sampling time for each foliage type. CK, SS, and DS represent no submergence, shallow submergence, and deep submergence, respectively.

Significant effects were observed in the degradation rate of cellulose and lignin based on foliage type (except for lignin degradation), water treatment, decomposition period, and the interactions of water treatment and decomposition time (*P* < 0.05) (Table 3). During decomposition, the cellulose and lignin degraded rapidly in the first 30 days of the decomposition period, although an apparent degradation of both cellulose and lignin was also observed in the following days after that (Figure 4). By the end of the one-year decomposition, the degradation of cellulose and lignin from all treatments ranged from 58.6 to 98.0% and from 79.9 to 93.8%, respectively. Cellulose showed a strong tendency to degrade when submerged in both the SS and DS treatments, with a significantly higher degradation of cellulose 12.8–29.7% in SS and 12.7–24.8% in DS than in CK, respectively. More specifically, the Chinese willow had the highest cellulose release rate in all three water treatments, reaching 5.57, 9.53, and 6.91 g⋅g^–1^⋅day^–1^ in CK, SS, and DS, respectively (Table 4). Likewise, the degradation rate of lignin in SS and DS treatments were also significantly greater than in CK. By the end of the decomposition, the lignin degradation of each foliage type was 9.5–13.2% significantly higher in SS and 6.3–9.2% significantly higher in DS, when compared with that in CK (Figure 4). The lignin degradation rate was the highest by mixtures under CK and DS treatments, reached 4.67 and 6.25 g⋅g^–1^⋅day^–1^, while under SS, it was the highest by bald cypress and reached 6.89 g⋅g^–1^⋅day^–1^ (Table 4). Very interestingly, all the three foliage types demonstrated the highest cellulose and lignin loss in SS among the three water treatments (except for mixed leaf litter of lignin) (Table 4). Throughout the experiment, the degradation of cellulose and lignin was significantly correlated with changes in the foliage chemical traits and the decomposition environmental factors that affected decomposition, such as concentrations of cellulose and lignin, and contents C, N, and P, as well as the temperature, water gradients, dissolved oxygen concentration, and electrical conductivity (Tables 5, 6).

**TABLE 4:** Changes in the cellulose and lignin degradation rates (k, g⋅g^–1^⋅day^–1^) of the leaves of bald cypress, Chinese willow, and the mixed-leaf samples under different water treatments.

Foliage type	Water treatment	Cellulose		Lignin	
		*k*′	R^2^	*k*′	R^2^
Bald cypress	CK	2.43	0.62	4.62	0.80
	SS	4.65	0.76	6.89	0.92
	DS	4.12	0.73	5.85	0.85
Chinese willow	CK	5.57	0.80	3.89	0.83
	SS	9.53	0.89	5.36	0.75
	DS	6.91	0.81	4.94	0.78
Mixtures	CK	4.07	0.84	4.67	0.89
	SS	7.66	0.89	5.78	0.80
	DS	6.42	0.85	6.25	0.79

*CK, SS, and DS represent no submergence, shallow submergence, and deep submergence, respectively. k′, k × 10^3^; all *P* < 0.001.*

**TABLE 5:** Regression model of the stepwise regression analysis between cellulose and lignin degradation rates (%/month) during one-year and initial chemical properties of foliar litter.

Degradation rate	Water treatment		Regression model				
				Step 1	Step 2	Step 3	Step 4
			a_0_	a_1_X_1_ (R^2^)	a_2_X_2_ (R^2^)	a_3_X_3_ (R^2^)	a_4_X_4_ (R^2^)
Cellulose	CK	=	109.720	−3.527 Cellulose (0.552)	−1.775 Lignin (0.676)	+1.842N (0.765)	−0.055C (0.774)
	SS	=	108.150	−1.872 Cellulose (0.551)	−0.769 Lignin (0.688)	−0.418N (0.712)	
	DS	=	114.074	−1.507 Cellulose (0.480)	−0.794 Lignin (0.625)		
Lignin	CK	=	104.949	−2.549 Lignin (0.593)	−11.180P (0.645)	+0.087C (0.674)	−0.967Cellulose (0.711)
	SS	=	131.761	−1.671 Lignin (0.729)	−0.652N (0.830)		
	DS	=	110.258	−1.461 Lignin (0.598)	0.045C (0.709)	−0.983N (0.739)	

**a*_1_, *a*_2_, *a*_3_, and *a*_4_ show regression coefficients. *X*_1_, *X*_2_, *X*_3_, and *X*_4_ show independent variables (litter chemical traits at each phase). Data in parentheses represent coefficients of determination (R^2^) at each step. CK, SS, and DS involve no submergence, shallow submergence, and deep submergence, respectively. C, total organic carbon; N, total nitrogen; P, total phosphorus; *a*_0_, intercept.*

**TABLE 6:** *F*-values for the regression analysis between cellulose and lignin degradation rate (%/month) and decomposition environment during one-year of foliage decomposition.

Water treatment	Foliage type	Cellulose				Lignin			
		T (°C)	WC (SQU^−1^)	pH	DO (mg⋅L^–1^)	T (°C)	WC (S⋅m^−1^)	pH	DO (mg⋅L^–1^)
CK	Bald cypress	(+)8.95**	(+)6.92*	(−)5.90*		(+)17.38***	(+)16.18***	(−)13.65**	
	Chinese willow	(+)26.43***	(+)23.40***	(−)21.54***		(+)13.21**	(+)12.81**	(−)7.35*	
	Mixtures	(+)5.93*	(+)9.83**	(−)9.251**		(+)7.49**	(+)117.7**	(−)10.34**	
SS	Bald cypress	(+)7.76**	(−)9.95**	0.57	(−)4.46*	(+)33.00***	(−)24.75***	(−)11.57**	(−)10.49**
	Chinese willow	(+)20.67***	(−)15.53***	3.99	(−)5.16*	(+)7.92**	(−)7.90**	1.40	1.98
	Mixtures	(+)13.70**	(−)12.64**	2.36	(−)3.70	(+)6.20*	4.10	0.44	0.36
DS	Bald cypress	1.54	(−)15.94***	0.18	0.13	2.36	(−)23.49***	0.12	0.67
	Chinese willow	(+)5.35*	(−)40.24***	1.92	0.01	(+)7.88**	(−)43.67***	(−)5.57*	0.45
	Mixtures	(+)5.85*	(−)42.14***	2.61	0.02	1.03	(−)14.11**	0.09	1.72

Significant and negative correlations are indicated by (+) and (−), respectively. CK, SS, and DS involve no submergence, shallow submergence, and deep submergence, respectively. *P < 0.05, **P < 0.01, ***P < 0.001. T, temperature; WC, water conductivity; DO, dissolved oxygen.

## Discussion

Water treatments significantly promoted the decomposition of various types of samples; approximately 79–90% of their initial mass was lost after one-year of decomposition in SS and DS environments (Figure 1). Cellulose and lignin degraded rapidly in the early phrase of decomposition (i.e., in the first 30 days) (Figure 4), while during the entire decomposition period, the degradation rates of cellulose and lignin were always greater under SS and DS than CK treatment (Table 4), which indicated that this type of degradation occurs much earlier and at high rates in a riparian ecosystem as compared with previous findings in terrestrial ecosystems (Thevenot et al., 2010; Berg and McClaugherty, 2014; Yue et al., 2016). Previous studies have shown that the microbial action in the decomposition process in relatively humid environments is rapidly enhanced within 30–90 days (Rejmánková and Houdková, 2006) and that a large number of extracellular enzymes that decompose lignin are secreted (Bugg et al., 2011). This showed that when various types of foliage were decomposed for 30 days, lignin was rapidly decomposed into small molecule compounds under the action of strong microorganisms (Brown and Chang, 2014). During this period, the release and return of organic C come mainly from the degradation of lignin. The rapid degradation of lignin in water also accelerates the degradation of cellulose, which confirmed our first hypothesis. However, the type of species was the main controlling factor for these processes, and the decomposition cycle and accompanying environmental factors also significantly regulated these processes (Table 3).

Previous studies on the dynamics of cellulose degradation in the decomposition of terrestrial ecosystems showed that the concentration as well as the mass of cellulose was continuously decreasing during the whole decomposition process (Li et al., 2016). Our research results on the cellulose concentration and mass dynamics were similar to those of terrestrial ecosystems (Figures 1, 2), but the degradation rates in water treatments (SS and DS) were much greater than those in the non-flooded environment treatment (CK) (Table 4). Compared with lignin, cellulose has a relatively simple structure, is highly biodegradable, and thus can decompose relatively rapidly (Berg and McClaugherty, 2014; He et al., 2015). During the early stages of decomposition, leaf litter was enriched with unprotected cellulose; hence, the rate of cellulose degradation was rapid until the unshielded portions were completely consumed. Moreover, although the degradation of lignin was much pronounced in the first 30 days, it is likely that the protected cellulose was partially broken, because degradation of cellulose was substantially influenced by the lignin concentration (Table 5).

Lignin is the most difficult complex to decompose in leaf litter as it is composed of complex, stable, and diverse amorphous three-dimensional macromolecules, hence with the slowest degradation rate of any cellular matter (Brown and Chang, 2014; Janusz et al., 2017). Generally, due to its structure, it is preserved, and the concentration usually increases in the early stages of foliage decomposition (Thevenot et al., 2010; Berg and McClaugherty, 2014). However, other studies have suggested that the bioavailability and decomposability of lignin are higher than previously thought, and experiments in terrestrial ecosystems have proven that lignin becomes strongly degraded in the first 200 days of decomposition, which is the new conceptual model of the degradation dynamics of litter lignin proposed by Klotzbucher et al. (2011). Similarly, Klotzbucher et al. (2011) and Yue et al. (2016), who studied the dynamics of lignin during the litter decomposition in a forest river, also found that lignin degraded in the initial phase of decomposition; both studies regarded the degradation of lignin as being closely related to the dissolved organic C in foliage. In our study, we found that the degradation pattern of lignin in the aquatic environment was also degraded at an early stage (especially the first 30 days of decomposition). This probably occurs in the early stage of decomposition because the availability of easily decomposed C sources is higher than that in the later stage, which is beneficial to the degradation of lignin. However, in the later incubation phases, after the loss of the easily degradable compounds, a marked decline in microbial activity was seen following the reduction in available nutrients (Berg and McClaugherty, 2014; Brown and Chang, 2014); therefore, the availability of bioavailable nutrients becomes a limiting factor (Klotzbucher et al., 2011). Thus, as foliage decomposition continues, the loss of lignin gradually slowed down (Figure 2). The rapid degradation of lignin in a riparian zone may prove that in another ecosystem, the bioavailability and degradability of lignin may indeed be higher than previously expected. Moreover, evidence revealed that during the early phase of foliage decomposition, soluble constituents such as cellulose in leaf litter help to maintain a rich microbial community (Cotrufo et al., 2013), which also further facilitate the process of lignin degradation. In addition, the cellulose and lignin degradation rates were shown to be primarily regulated by the changes in chemical traits, such as the concentrations of cellulose and lignin, and the C, N, and P contents (Table 5), which are also the basic nutrients required by microbial activities (Gessner et al., 2010; Berg and McClaugherty, 2014).

In addition to foliage type and decomposition time, local environmental factors (such as temperature, water gradients, and dissolved oxygen) have also been found to significantly affect the degradation of cellulose and lignin (Couteaux et al., 1995; Fierer et al., 2005; Xie et al., 2015; Martínez et al., 2016; Moghadam and Zimmer, 2016), which was also consistent with our study (Table 6). The difference in the range of temperature and moisture in different decomposition environments may also be one of the reasons for the difference in degradation rates of cellulose and lignin for each foliage type. Besides, the buffering of the water environment may cause the temperature of the water body to fluctuate very little in different periods, and the continuous supply of upstream nutrients will guarantee the microbial community will remain active (Martínez et al., 2014). The SS environment combined rapid water flow velocity with higher temperatures and greater concentrations of dissolved oxygen (Table 2). These conditions themselves can lead to faster decomposition and abundant microbial activity, and many types of microbial activity in turn cause significant fragmentation of the litter’s physical structure (Berg and McClaugherty, 2014), resulting in the degradation rate of cellulose and lignin in SS that was faster than that in DS treatment (except for mixed litter lignin degradation), which also confirms our second hypothesis. These results indicate that regional environmental factors have an important effect on cellulose and lignin degradation, whether it is a direct or indirect effect of biotic or abiotic conditions; and the difference in environmental factors represented by different water treatments and different decomposition stages had a relatively prominent effect on cellulose and lignin degradation (Table 6).

## Conclusion

Investigating the degradation dynamics of foliar cellulose and lignin in dominant species of trees used in reforestation within the context of riparian environments is of great significance because it provides ideas for improving the effective management of recalcitrant substrates in riparian ecosystems. The present study in the riparian zone of the TGDR area revealed that water gradients accelerated the degradation rates of cellulose and lignin of each foliage type, and foliage degradation was also significantly affected by the concentrations cellulose and lignin; C, N, and P contents; and other environmental factors. Thus, our research provides helpful information for the scientific management of plantations associated with recalcitrant material cycling to establish a sustainable riparian protection forest in the TGDR area.

## Data Availability Statement

The raw data supporting the conclusions of this article will be made available by the authors, without undue reservation.

## Author Contributions

CL, ZC, and CW conceived the study, designed the experiments, and supervised the entire study. ZC and XC performed the experiments. CL and ZC wrote the manuscript. All authors contributed to the article and approved the submitted version.

## Conflict of Interest

The authors declare that the research was conducted in the absence of any commercial or financial relationships that could be construed as a potential conflict of interest.
